# Syphilis

**Published:** 1888-04

**Authors:** 


SYPHILIS. By Jonathan Hutchinson, F. R. S., LL. D., Consulting Surgeon to the London Hospital, etc. Philadelphia: Lea Brothers & Co.

The name of Jonathan Hutchinson on the title-page of a work on syphilis is a sufficient guarantee of the excellence of the book. The present instance is no exception to this statement; the book is fully up to the author's great reputation. The first part of the book is devoted to a description of the disease from the initial lesion to and through its tertiary stage. The greater part of the book is taken up by clinical commentaries and illustrative cases, giving the reader a much more practical acquaintance with the protean manifestations of syphilis than he could acquire from a more didactic style of writing. These commentaries are short essays on every phase of syphilis, discussing, among other things, syphilis in relation to marriage, hereditary transmission, differential diagnosis, treatment of syphilis, etc.

Otis believes a solution of continuity necessary for the implantation of the syphilitic virus. Hutchinson believes that while abrasions of the surface favor contagion, they are by no means necessary. The author states his position on the etiology of syphilis in no uncertain language when he says: "The creed which will be found to interfuse not only this work, but almost all that I have ever written on syphilis, is that the disease depends upon a living and specific microbe. . . Considering the successes which the study of bacteriology has attained of late years, it is certainly surprising that no one has as yet been able to demonstrate the special microbe of syphilis. That this discovery is in reserve for some future investigator, I have the utmost confidence in believing."

The subject of treatment is exhaustively discussed; in regard to the primary lesion, the actual cautery or total excision is admissible if the chancre is not much indurated, or has not existed very long. We need not, however, expect to abort the disease by this measure, for the poison has already passed into the lymphatic system and possibly into the blood as well. Mr. Hutchinson thinks the best plan is to give ether and thoroughly cauterize the sore with the Paquelin cautery.

In the secondary and tertiary stages mercurials and the iodide of potash still stand unrivalled. The different methods of giving them are carefully stated.

The book is enriched by eight colored plates illustrative of various syphilitic lesions; these are well executed and are remarkably life-like.

